# Self-reported attention and hyperactivity symptoms among adults with epilepsy

**DOI:** 10.1055/s-0044-1779298

**Published:** 2024-01-29

**Authors:** Eduardo de Novaes Costa Bergamaschi, Gabriela Machado, Gabriel Martins Rodrigues, Katia Lin

**Affiliations:** 1 Universidade Federal de Santa Catarina, Florianópolis SC, Brazil.

**Keywords:** Epilepsy, Attention Deficit Disorder with Hyperactivity, Mental Disorders, Epilepsia, Transtorno do Deficit de Atenção com Hiperatividade, Transtornos Mentais

## Abstract

**Background** Patients with epilepsy (PWE) frequently have comorbid psychiatric disorders, the most common of which are depression and anxiety. Attention deficit disorder with hyperactivity (ADHD) is also more frequent among PWE, though that condition has been scarcely studied among the adult PWE population.

**Objective** This study aimed to compare the presence of ADHD symptoms between adult PWE and the general population.

**Methods** This was an observational case-control study. Ninety-five adult PWE from a tertiary center in southern Brazil were compared with 100 healthy controls. All subjects were submitted to three structured scales: 1) the World Health Organization Adult ADHD Self-Report Scale version 1.1 (ASRS); 2) the Hospital Anxiety and Depression Scale (HADS); and 3) the Adverse Events Profile (AEP). Dichotomic variables were analyzed through chi-square test and Fisher's exact test, as appropriate, and non-parametric variables were analyzed through the Mann-Whitney *U* test.

**Results** Medians and interquartile ranges (IR) were: 1) ASRS: 26.00 (IR: 18 to 38) among PWE versus 17.00 (IR: 11 to 24) among controls, *p* < 0.001; 2) HADS: 14.00 (IR: 8 to 21) among PWE versus 11.00 (IR: 8 to 16) among controls, *p* = 0.007; 3) AEP: 3800 (IR: 31 to 49) among PWE versus 33.00 (IR: 23 to 43) among controls, *p* = 0.001.

**Conclusion** PWE showed a higher burden of symptoms of ADHD, depression, and anxiety when compared with controls, which replicates in the Brazilian population the findings of current literature that point toward a higher prevalence of such disorders among PWE.

## Resumo

**Antecedentes** Pacientes com epilepsia (PCE) frequentemente apresentam comorbidades psiquiátricas, principalmente depressão e ansiedade. O transtorno do déficit de atenção e hiperatividade (TDAH) também é mais frequente nos PCE, porém foi pouco estudado na população adulta de PCE.

**Objetivo** Comparar a presença de sintomas de TDAH entre PCE adultos e a população geral.

**Métodos** Noventa e cinco PCE adultos de um centro terciário no Sul do Brasil foram comparados a 100 controles saudáveis. Todos os sujeitos foram submetidos a três escalas estruturadas: 1) a Escala Autorrelatada de TDAH em Adultos da Organização Mundial da Saúde, versão 1.1 (ASRS); 2) a Escala Hospitalar de Ansiedade e Depressão (HADS); e 3) o Perfil de Eventos Adversos (AEP). Variáveis dicotômicas foram analisadas através dos testes chi-quadrado e exato de Fisher, conforme apropriado, e as variáveis não paramétricas foram analisadas através do teste *U* de Mann-Whitney.

**Resultados** As medianas e os intervalos interquartis (IIQ) foram: 1) ASRS: 26.00 (IIQ: 18 a 38) em PCE *versus* 17.00 (IIQ: 11 a 24) nos controles, *p* < 0,001; 2) HADS: 14.00 (IIQ: 8 a 21) em PCE *versus* 11.00 (IIQ: 8 a 16) nos controles, *p* = 0,007; 3) AEP: 38.00 (IIQ: 31 a 49) em PCE *versus* 33.00 (IIQ: 23 a 43) nos controles, *p* = 0,001.

**Conclusão** PCE apresentaram uma maior carga de sintomas de TDAH, depressão e ansiedade quando comparados aos controles, o que replica na população brasileira os achados da literatura atual, que apontam para uma maior prevalência de tais transtornos entre PCE.

## INTRODUCTION

Adults with active epilepsy often have comorbid disorders. 1 2 3 Previous reports have found a higher prevalence of dementia, Parkinson's disease, migraine, chronic fatigue, stroke, heart disease, chronic bronchitis, asthma, peptic ulcers, gastrointestinal bleeding, diabetes, arthritis, and other somatic disorders among patients with epilepsy (PWE) when compared with the general population. 2 PWE are also at higher risk of psychiatric comorbidities, including mood, anxiety, psychotic, and attention deficit disorders. 3 4 Current evidence points toward a bilateral relationship between epilepsy and psychiatric disorders. 3

The most prevalent psychiatric disorder in PWE is depression, especially among patients with poor seizure control, with prevalence rates ranging from 21 to 33% among PWE with uncontrolled seizures and from 4 to 6% among seizure-free patients. 3 PWE also display a higher suicide rate when compared with the general population. 3 Anxiety disorders are also frequently comorbid with epilepsy, with an estimated prevalence between 15 and 25%. 3

Attention deficit disorder with hyperactivity (ADHD) is defined according to the Diagnostic and Statistical Manual of Mental Disorders, Fifth Edition (DSM-5) as a neurodevelopmental disorder characterized by a pervasive pattern of inatention and/or hyperactivity/impulsivity that impacts functionality and development. 5 Clinical manifestations of ADHD usually begin during childhood but may persist into adulthood, and some evidence suggests they may have a late onset during adulthood in some patients. 6 In Brazil, a cohort study found that 12.2% of adults at 18 to 19 years of age fulfilled the DSM-5 criteria for ADHD minus the age of onset criteria. 7 An association between ADHD and epilepsy in the pediatric population was reported both by populational and tertiary center studies. 8 9 10 11 The prevalence of ADHD among adult PWE has been less studied, though some studies have reported a frequency of symptoms of ADHD of approximately one-fifth among adult PWE. 12 13 ADHD symptoms were associated with a higher burden of depression and anxiety symptoms in the adult PWE population. 13 No studies investigating the association of epilepsy and ADHD symptoms among Brazilian adult PWE have been published.

Psychiatric comorbidities among PWE are associated with poor quality of life, lower odds of seizure control, higher risks of drug side effects, and premature mortality. 4 Alternatively, psychiatric comorbidities increase the burden of epilepsy and its associated health costs. 4 Therefore, it is important to assess and treat psychiatric disorders associated with epilepsy.

This study aimed to compare the presence of ADHD symptoms between adult PWE in an outpatient tertiary center in southern Brazil and the general population, as well as investigate potential associations between ADHD, depression, and anxiety symptoms and correlate psychiatric symptoms with anti-seizure drugs (ASD) adverse effects and drug load in the adult PWE population.

## METHODS

This was an observational case-control study. For the case group, ninety-five consecutive adults with epilepsy followed at the specialized epilepsy outpatient clinic of the University Hospital of the Federal University of Santa Catarina ( *Hospital Universitário da Universidade Federal de Santa Catarina* – HU-UFSC). All patients were 18 years or older and had well-defined epilepsy diagnoses according to the International League Against Epilepsy (ILAE) criteria. 14 Patients with moderate or severe mental disability and those with suspected psychogenic non-epileptic seizures were excluded. The control group included 100 non-epileptic consecutive persons who accompanied patients during consultations or hospital stays at HU-UFSC. To be included in the control group, the subject should not have any known current or past neurological or psychiatric condition. Clinical data of case subjects were collected from their medical records and included epilepsy type, epileptic syndrome, and seizure frequency (epileptic paroxysms per month). Case and control groups were compared according to age and sex.

Three validated scales previously adapted to Brazilian Portuguese were applied to all subjects: 1) the World Health Organization Adult ADHD Self-Report Scale version 1.1 (ASRS) 15 16 ; 2) the Hospital Anxiety and Depression Scale (HADS) 17 ; and 3) the Adverse Events Profile (AEP). 18 19 20 Though said scales are usually self-administered, in this study they were read aloud to the subjects by the researchers to homogenize their application between illiterate and non-illiterate subjects. Data were collected from January 2012 to December 2013.

ASRS consists of 18 items based on symptoms listed in criterion A for ADHD from the Diagnostic and Statistical Manual of Mental Disorders, Fourth Edition (DSM-IV), 21 and adapted to adults. 15 16 Each item is scored according to the frequency of each symptom over the previous 6 months, ranging from 0 (never) to 4 (very often). 15 16 It is divided into 2 parts, each with 9 items: Part A, which assesses symptoms of inattention, and Part B, which assesses hyperactivity and impulsiveness. 15 16 A total score above 24 is considered strongly suggestive of ADHD. 15 16 A positive screening for 8 items of the scale has been appointed as the optimal cut-off to discriminate between persons with ADHD from controls. 16 ASRS was adapted to Brazilian Portuguese in 2006. 15

HADS includes 14 items, 7 of which assess anxiety symptoms (HADS-A) and 7 focused on depressive symptoms (HADS-D). 17 One point is scored for each present symptom, and total scores of 8 or over are considered positive. 17

AEP was designed to evaluate the presence of adverse effects of ASD. 18 19 20 It comprises 19 items corresponding to signs and symptoms frequently attributed to ASD use. 18 19 20 The respondent must report the severity of signs and symptoms experienced over the previous four weeks, which is scored from 1 (not a problem) to 4 (very serious problem) for each symptom. 18 19 20 AEP was applied to control subjects to evaluate the possibility that said scale could misidentify symptoms attributable to conditions unrelated to ASD or epilepsy as secondary to ASD usage among PWE.

The total scores of patients and controls in each scale were compared. Further comparison was made between the scores of both groups in Parts A and B of ASRS and parts HADS-A and HADS-D of HADS. A bivariate correlation analysis between the three scales was also conducted.

To assess the potential influence of ASD usage on symptom reporting, we conducted a correlation analysis between each score and total drug load. Total drug load was defined as the ratio between the prescribed daily ASD dose (PDD) and the defined daily dose according to WHO (DDD). 22 The defined daily dose is the average maintenance daily dose for each ASD by analysis of literature and drug registration data. 22 When multiple ASD were used by one patient, an individual PDD/DDD ratio was calculated for each drug. The total drug load was established as the average of all the individual ratios for said patient.

Statistical analysis was conducted with SPSS version 28.0 (SPSS Inc. Chicago, IL, USA). Dichotomic variables are expressed as total number and percentage, and continuous variables are expressed as mean ± standard deviation (SD). For hypothesis testing, dichotomic variables were analyzed through chi-square test and Fisher's exact test, as appropriate, and non-parametric variables were analyzed through the Student's *t* -test or Mann-Whitney *U* test, as appropriate. The Kolmogorov-Smirnov test was employed as a normality test. For correlation analyses, Spearman's correlation test was used. Post-hoc analyses were conducted to evaluate the eventual influences of age and sex over score results. A value of *p* under 0.050 was considered statistically significant.

This study was executed according to the Declaration of Helsinki and was approved by UFSC's research ethics committee (CAAE project number: 46928515.0.0000.0121). All subjects signed an informed consent form.

## RESULTS

Demographic and clinical characteristics of the total sample are shown in Table 1 . The control group was older than the case group and had a significantly larger female sample. In the case group, 66 (69.5%) patients were diagnosed with temporal lobe epilepsy (TLE), 7 (7.4%) with frontal lobe epilepsy (FLE), 4 (4.2%) with parietal lobe epilepsy (PLE), 5 (5.3%) with occipital lobe epilepsy (OLE), 6 (6.3%) with juvenile myoclonic epilepsy (JME), 2 (2.1%) with childhood absence epilepsy/juvenile absence epilepsy (CAE/JAE), and 5 (5.3%) with generalized epilepsy with tonic-clonic seizures (GETCS). According to epileptic etiology, 13 (13.7%) cases had genetic epilepsy, 27 (28.4%) had structural or metabolic epilepsy, and 55 (57.9%) had cryptogenic epilepsy.

**Table 1:** Demographic and clinical characteristics of case and control groups ( *n* = 195)

Characteristics		Case group ( *n* = 95)	Control group ( *n* = 100)	*p*
Age *		36.00 (26 to 52)	42.50 (32 to 53)	0.021 ^a^
Sex	Male	46 (48.4%)	30 (30.0%)	0.012 ^b^
	Female	49 (51.6%)	70 (70.0%)	
Epileptic etiology	Genetic	13 (13.7%)	n/a	n/a
	Structural/metabolic	27 (28.4%)	n/a	n/a
	Cryptogenic	55 (57.9%)	n/a	n/a
Seizure type	Generalized	12 (12.6%)	n/a	n/a
	Focal	83 (87.4%)	n/a	n/a

Notes: *Shown as “median (interquartile range)”; ^a^ Mann-Whitney U test; ^b^ Chi-square test.

The Kolmogorov-Smirnov test indicated that our sample's variables were not normally distributed. Therefore, the Mann-Whitney *U* test and Spearman's correlation test were employed.

The correlation analyses ( Table 2 ) showed that all three scales correlated positively. Total drug load showed a positive correlation with AEP, but not with the other scales. The frequency of focal onset aware seizures (FOAS) correlated positively with ASRS, AEP, HADS, and HADS-A; the frequency of focal onset impaired awareness seizures (FOIAS) correlated positively with AEP; the frequency of focal to bilateral tonic-clonic seizures (FBTCS) correlated positively with AEP, HADS, and HADS-D; and the frequency of generalized onset tonic-clonic seizures (GOTCS) correlated positively with HADS-D (data not shown). The frequency of absence seizures (AS) and myoclonic seizures (MS) showed no correlation with any of the scales (data not shown).

**Table 2:** Correlation analyses results (Spearman's correlation test; *n* = 195)

	Spearman's rho	*p*
ASRS x AEP*	0.576	< 0.001
ASRS x HADS*	0.546	< 0.001
AEP x HADS*	0.579	< 0.001
TDL x ASRS**	0.133	0.200
TDL x AEP**	0.207	0.044
TDL x HADS**	0.193	0.061

Abbreviations: AEP, Adverse Events Profile; ASRS, World Health Organization Adult Attention Deficit and Hyperactivity Disorder Self-Report Scale; HADS, Hospital Anxiety and Depression Scale; TDL, total drug load (defined as the ratio between prescribed daily dose of anti-seizure drugs and defined daily dose of anti-seizure drugs).

Notes: * *n* = 195 (case and control groups); ** *n* = 95 (case group).

The case group scored higher than the control group on all three scales ( Table 3 ). The scores of the case group were also higher on Parts A and B of ASRS and on part D of HADS. In the case group, patients with FLE showed lower median HADS-A scores when compared with other epileptic syndromes (5.00 versus 9.00, *p* = 0.032) Other individual epileptic syndromes did not differ from the others in any scale (data not shown).

**Table 3:** Self-report scale scores by study group ( *n* = 195)

	Cases*	Controls*	*p* **
ASRS	26.00 (18 to 38)	17.00 (11 to 24)	< 0.001
Part A	12.00 (8 to 20)	7 (3 to 11)	< 0.001
Part B	14.00 (8 to 18)	10 (6 to 14)	< 0.001
AEP	38.00 (31 to 49)	33.00 (23 to 43)	0.001
HADS	14.00 (8 to 21)	11.00 (8 to 16)	0.007
HADS-A	8.00 (5 to 13)	7.00 (4 to 10)	0.055
HADS-D	6.00 (3 to 10)	4.00 (2 to 6)	0.003

Abbreviations: AEP, Adverse Events Profile; ASRS, World Health Organization Adult Attention Deficit and Hyperactivity Disorder Self-Report Scale; HADS, Hospital Anxiety and Depression Scale; HADS-A, part of HADS assessing anxiety; HADS-B, part of HADS assessing depression.

Notes: *Data shown as “median (interquartile range)“; **Mann-Whitney U test.

Since case and control groups had statistically significant age and sex differences, post-hoc analyses were conducted to evaluate eventual influences of age and sex over score results. Female subjects had higher HADS scores than their male counterparts, as well as higher scores on both sub-scores of HADS ( Table 4 ). Age correlated negatively with the scores on ASRS, ASRS Part A, ASRS Part B, AEP, HADS, and HADS-A ( Table 5 ).

**Table 4:** Total sample (case and control groups) self-report scale scores by sex ( *n* = 195)

	Male*	Female*	*p* **
ASRS	21.00 (13 to 36)	21.00 (13 to 30)	0.304
Part A	10.50 (5 to 18)	9.00 (5 to 15)	0.189
Part B	12.00 (6 to 17)	11.00 (6 to 17)	0.612
AEP	34.00 (29 to 42)	37.00 (25 to 48)	0.159
HADS	10.00 (6 to 16)	13.00 (8 to 21)	0.009
HADS-A	6.50 (3 to 11)	8.00 (5 to 12)	0.040
HADS-D	3.50 (2 to 6)	5.00 (3 to 9)	0.003

Abbreviations: AEP, Adverse Events Profile; ASRS, World Health Organization Adult Attention Deficit and Hyperactivity Disorder Self-Report Scale; HADS, Hospital Anxiety and Depression Scale; HADS-A, part of HADS assessing anxiety; HADS-B, part of HADS assessing depression.

Notes: *Data shown as “median (interquartile range)”; **Mann-Whitney U test.

**Table 5:** Total sample (case and control groups) correlation between age and self-reported scales (Spearman's correlation test; *n* = 195)

Age x	Spearman's rho	*p*
ASRS	-0.178	0.013
Part A	-0.149	0.037
Part B	-0.158	0.028
AEP	-0.238	0.003
HADS	-0.153	0.032
HADS-A	-0.179	0.012
HADS-D	-0.107	0.137

Abbreviations: AEP, Adverse Events Profile; ASRS, World Health Organization Adult Attention Deficit and Hyperactivity Disorder Self-Report Scale; HADS, Hospital Anxiety and Depression Scale; HADS-A, part of HADS assessing anxiety; HADS-B, part of HADS assessing depression.

## DISCUSSION

Most PWE in this study had focal epilepsy, and the most frequent etiology was cryptogenic epilepsy. Though the case group showed a balanced male-to-female ratio, there was a significant predominance of female subjects in the control group. Since the control group was recruited from people accompanying patients during consultations or hospital stays, unequal distribution was expected, for evidence points to a female predominance among inpatients' caregivers in Brazil. 23 Females in our sample showed higher scores on HADS, and the predominance of females in the control group may have attenuated the observed difference in HADS scores between PWE and controls. ASRS scores were nominally higher among males in our sample but without statistical significance. Alternatively, higher ASRS scores among males have been reported in the literature. 24 25

ASRS, HADS, and AEP correlated positively, but only AEP showed a correlation with total drug load among PWE. The association between ADHD symptoms and anxiety and depression among adult PWE has already been reported. 13 The absence of correlation between both ASRS and HADS with total drug load may indicate that the higher burden of ADHD, anxiety, and depression symptoms among PWE are not mere adverse effects of ASD but rather reflect common pathophysiological substrates underlying epileptogenesis and comorbid psychiatric disorders. The possibility that cognitive and behavioral disorders associated with epilepsy may be multifactorial in origin has been noted in the literature. 26 As expected, AEP showed a positive correlation with total drug load. The positive correlation between the frequency of several seizure types and ASRS, AEP, and HADS was also expected.

PWE in this sample showed higher scores on ASRS, HADS, and AEP when compared with controls. Although it is possible that some control subjects could have undiagnosed psychiatric conditions, a statistically significant difference of symptoms of ADHD, depression, and anxiety between PWE and controls was nonetheless found, suggesting PWE are at greater risk of psychiatric comorbidities. The higher frequency of symptoms of ADHD, depression, and anxiety among PWE is in accordance with the existing scientific literature, in which the presence of epilepsy has been associated with a higher prevalence of such psychiatric comorbid disorders than that observed in the general population. 3 4 8 9 10 11 12 13 16 26 27 28 29 30 31 A history of epilepsy and/or febrile seizures during childhood has been linked to an increased risk of developing ADHD. 32 Albeit ADHD is more frequent among the pediatric population with epilepsy, adult PWE have also shown a higher prevalence of ADHD when compared with the general population, 12 13 16 and our finding of a higher ADHD symptoms burden among PWE is consistent with the current literature and replicates its findings in the Brazilian population.

Regarding differences in scores between different epileptic syndromes, we found that subjects with FLE scored lower on HADS-A in comparison to other epileptic syndromes, though no association between ADHD symptoms and specific epileptic syndromes was found. It is possible that differences in epileptogenesis, neurobiology, and ASD use between distinct epileptic syndromes may explain the lower scores on HADS-A observed among patients with FLE in our sample. One study reported a higher frequency of ADHD symptoms among women with JME compared with other genetic generalized epilepsies. 33 Another research did not find differences in the prevalence of psychiatric comorbidities between patients with focal versus generalized epilepsy. 29 Additionally, there is evidence of selective impairment in selective attention, divided attention, and set-shifting in persons with TLE. 34 A higher prevalence of hyperactivity has been reported among carriers of a mutation of the leucine-rich glioma inactivated 1 (LGI1) gene, which has been linked to autosomal dominant TLE. 35 One study found a 5-fold increase in psychostimulant use among patients with refractory epilepsy, 36 while another identified a higher prevalence of ADHD among patients with psychogenic non-epileptic seizures. 28

This study has some limitations. Due to the impracticality of conducting complete and comprehensive psychiatric evaluations for the subjects in the control group, the assessment of psychiatric symptoms was conducted exclusively based on structured scales, and the study protocol did not include clinical evaluation of psychiatric symptoms, precluding the establishment of formal clinical diagnoses of ADHD, depression and/or anxiety in our sample. Therefore, we could not assess the actual prevalence of said disorders in our study sample. Alternatively, we could not exclude previous or current psychiatric conditions among PWE. The recruitment of people without any known current or past neurological or psychiatric disorder for the control group is a potential confounding factor since ADHD usually begins during childhood and adolescence, and excluding previously diagnosed ADHD patients may have artificially reduced the burden of ADHD symptoms in the control group. Finally, the symptoms of ADHD, depression, and anxiety may overlap, and the employed scales may not precisely differentiate between those psychiatric conditions. 37 38 39

In conclusion, adult PWE show a higher burden of ADHD symptoms when compared with the general population. Symptoms of ADHD are positively associated with depression and anxiety symptoms, which are also more frequent among PWE. Future studies on ADHD symptoms in the adult PWE population may benefit from the inclusion of clinical evaluation of psychiatric symptoms in the study protocol to assess the true prevalence of ADHD among adult PWE.
